# Structural basis for divergent C–H hydroxylation selectivity in two Rieske oxygenases

**DOI:** 10.1038/s41467-020-16729-0

**Published:** 2020-06-12

**Authors:** April L. Lukowski, Jianxin Liu, Jennifer Bridwell-Rabb, Alison R. H. Narayan

**Affiliations:** 1 Program in Chemical Biology, University of Michigan, Ann Arbor, MI USA; 2 Life Sciences Institute, University of Michigan, Ann Arbor, MI USA; 3 Department of Chemistry, University of Michigan, Ann Arbor, MI 48109 USA

**Keywords:** X-ray crystallography, Biocatalysis

## Abstract

Biocatalysts that perform C–H hydroxylation exhibit exceptional substrate specificity and site-selectivity, often through the use of high valent oxidants to activate these inert bonds. Rieske oxygenases are examples of enzymes with the ability to perform precise mono- or dioxygenation reactions on a variety of substrates. Understanding the structural features of Rieske oxygenases responsible for control over selectivity is essential to enable the development of this class of enzymes for biocatalytic applications. Decades of research has illuminated the critical features common to Rieske oxygenases, however, structural information for enzymes that functionalize diverse scaffolds is limited. Here, we report the structures of two Rieske monooxygenases involved in the biosynthesis of paralytic shellfish toxins (PSTs), SxtT and GxtA, adding to the short list of structurally characterized Rieske oxygenases. Based on these structures, substrate-bound structures, and mutagenesis experiments, we implicate specific residues in substrate positioning and the divergent reaction selectivity observed in these two enzymes.

## Abstract (web summary)

Rieske oxygenases are iron-dependent enzymes that catalyse C–H mono- and dihydroxylation reactions. Here, the authors characterise two cyanobacterial Rieske oxygenases, SxtT and GxtA that are involved in the biosynthesis of paralytic shellfish toxins and determine their substrate free and saxitoxin analog-bound crystal structures and by using mutagenesis experiments identify residues, which are important for substrate positioning and reaction selectivity.

## Introduction

**Fig. 1:**
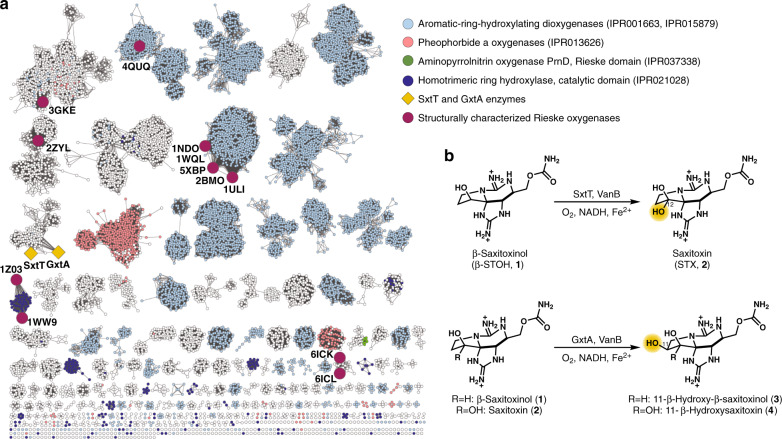
Extent of characterization of the Rieske oxygenase enzyme superfamily. **a** Sequence similarity network of Rieske oxygenases (Pfam PF00355) generated by the EFI-EST^59^ server at an E-value of 5 and alignment score of 65, visualized in Cytoscape v3.7.1^60^. The network shown was trimmed to include only Rieske proteins that contain the non-heme iron catalytic domain (IPR017941, IPR005805, and IPR domains listed in the figure). PDB codes represent the first structure determined for each enzyme. 1ULI, biphenyl dioxygenase; 5XBP, nitroluene dioxygenase; 1NDO, naphthalene dioxygenase; 1WQL, cumene dioxygenase; 2BMO, nitrobenzene dioxygenase; 1WW9, carbazole 1,9α-dioxygenase; 1Z03, 2-oxoquinoline 8-monooxygenase; 3GKE, dicamba monooxygenase; 4QUQ, stachydrine demethylase; 2ZYL, 3-ketosteroid-9-α-hydroxylase; 6ICK, NdmA; 6ICL, NdmB. **b** Rieske monohydroxylases SxtT and GxtA catalyze highly selective C–H functionalization reactions in paralytic shellfish toxin biosynthesis.

Enzymes that perform C–H hydroxylation reactions often demonstrate impressive control over site- and stereoselectivity on complex scaffolds^1–3^. This precision is emulated in numerous natural product biosynthetic pathways by several classes of metalloenzymes including cytochrome P450 monooxygenases^4^, non-heme α-ketoglutarate-dependent oxygenases^5^, and Rieske oxygenases^6^. To accomplish selective modification of inert C–H bonds, these enzyme classes employ molecular oxygen to generate high-valent iron intermediates, which activate a substrate for hydroxylation through hydrogen atom abstraction^7–11^. Examples include the Fe(IV)-oxo and Fe(III)-hydroperoxo intermediates employed by heme-containing systems^12,13^ and the Fe(III)-superoxo^14^, Fe(IV)-oxo, or proposed Fe(V)-oxo species used by non-heme systems^7^. In the Rieske oxygenase class, work to date has been conducted on a small sample of enzymes, specifically those that perform dioxygenation of aromatic rings. However, enzymes in this class catalyze a plethora of reactions, including monohydroxylation^15,16^, dihydroxylation^17^, dealkylation of heteroatoms^18^, desaturation^19^, and cyclization reactions^20^. The sequence divergence within the class presents a challenge in assigning one of these functions to a given Rieske oxygenase, let alone predict the substrate, site of reactivity, or type of reaction (Fig. 1a).

Structural studies on enzymes with divergent selectivities have the potential to illuminate critical features that are responsible for selectivity and provide valuable insight on how reactivity can be controlled in biocatalytic reactions. Rieske oxygenases in particular are underexplored in this regard, with only twelve available structures of different enzymes in the Protein Data Bank^21^. Five of these enzymes, including biphenyl dioxygenase^22–25^, 3-nitrotoluene dioxygenase^26^, naphthalene 1,2-dioxygenase^27–30^, cumene dioxygenase^31^, and nitrobenzene dioxygenase^32^, are clustered together in the sequence similarity network (SSN) presented in Fig. 1a (light blue) and catalyze the oxidative degradation of aromatic compounds (IPR001663, IPR015879). The remaining seven structurally elucidated Rieske oxygenases are also involved in degradative pathways: carbazole 1,9α-dioxygenase^33^, 2-oxoquinoline 8-monooxygenase^34^, dicamba monooxygenase^35,36^, stachydrine demethylase (2ZYL), and NdmA/NdmB^37^. These enzymes each degrade aromatic heterocycles and/or phenols, whereas 3-ketosteroid-9-α-hydroxylase (KshA^15^) catabolizes the cycloalkane rings of ketosteroids. The disparity between the large number of known Rieske oxygenases and the number that have been biochemically or structurally investigated highlights the need for further studies on the vast number of uncharacterized Rieske oxygenases.

In this work, we focus on two cyanobacterial Rieske oxygenases, SxtT and GxtA, that are involved in the biosynthesis of paralytic shellfish toxins (PSTs, Fig. 1b)^16^. PST natural products exhibit high affinities for neurotoxin receptor site 1 of human voltage-gated sodium channels (VGSCs). The affinity for VGSCs varies based on the pattern of functional groups present on the PST tricyclic core^38,39^. Recently, we demonstrated that SxtT and GxtA are responsible for complementary site- and stereoselective C–H hydroxylation reactions on the PST scaffold (Fig. 1b)^16^. We discovered that SxtT operates on a number of tricyclic substrates, preferring β-saxitoxinol (β-STOH, **1**), to install the C12 α-hydroxyl group, generating the potent natural product, saxitoxin (STX, **2**, Fig. 1b)^16^. GxtA, which shares 88-percent sequence identity with SxtT (Supplementary Fig. 1), displays divergent selectivity. GxtA hydroxylates the C11 β-position of β-STOH (**1**) and STX (**2**) to afford 11-β-hydroxy-β-saxitoxinol (**3**) and 11-β-hydroxysaxitoxin (11-β-hydroxySTX, **4**), respectively (Fig. 1b). Herein, we report crystal structures of SxtT and GxtA. Using the determined SxtT, GxtA, and STX analog-bound structures coupled with biochemical assays, we find that the impeccable site-selectivity of the SxtT catalyzed reaction is in part dictated by the formation of a hydrogen bond in the active site between the guanidinium moiety of the substrate and a polar residue at position 276. In GxtA, we instead find that the guanidinium moiety of the substrate is positioned by Gln226 and Asp239 residues. We identify a flexible loop that may play a role in gating access of substrate to an active site tunnel and speculate that changes to this region of the proteins also play important roles in reaction selectivity. Taken together, this work provides the structural basis for site selectivity of two Rieske monooxygenases and facilitates targeted engineering efforts to tune selectivity for the generation of non-natural or synthetically intractable PST analogs for VGSC studies.

## Results

### SxtT and GxtA showcase structurally similar trimeric architectures

**Fig. 2:**
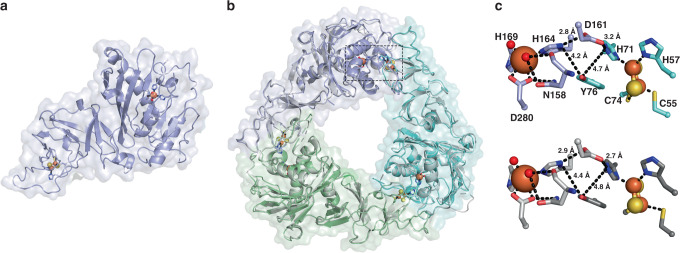
SxtT and GxtA showcase structurally similar trimeric architectures and metallocenter arrangements. **a** The monomeric unit of SxtT reveals that the metallocenters are located approximately 45-Å apart. **b** The trimeric quaternary structures of SxtT (colors) and GxtA (gray) are overlaid with one another. The trimeric arrangement of subunits allows for the close positioning of the metallocenters. **c** Two residues, Asp161 and Tyr76, which are found at the subunit-subunit interface of both SxtT (top panel) and GxtA (bottom panel, same residue numbering as SxtT) bridge the Rieske cluster to the non-heme iron center. SxtT is shown in purple and cyan and GxtA is gray.

The crystal structure of SxtT was determined to 1.86 Å resolution using a modified model of dicamba monooxygenase for molecular replacement (SxtT and dicamba monooxygenase share 31-percent sequence identity with one another). The structure of SxtT was subsequently used to solve the 2.10 Å resolution structure of SxtT with a STX analog bound and the 2.20 Å resolution structure of GxtA (Supplementary Table 3). The latter was then used to solve the 2.18 Å resolution structure of substrate-analog bound GxtA (Supplementary Table 3). As predicted based on the high sequence identity of SxtT and GxtA, the proteins are structurally similar with a root-mean-square deviation (r.m.s.d) determined by PyMOL of 0.971 Å over 950 Cα atoms. In accordance with other characterized Rieske oxygenases, SxtT and GxtA are composed of two domains that make up the catalytic α-subunit: an N-terminal Rieske domain, and a C-terminal catalytic iron-containing domain (Fig. 2a). In a single monomer, the Rieske cluster and the mononuclear iron site are located nearly 45 Å apart, a distance that is too far to permit intra-subunit electron transfer (Fig. 2a). As observed in other Rieske oxygenases, the monomeric subunits of SxtT and GxtA arrange into an α3 trimeric architecture to facilitate inter-subunit electron transfer from the Rieske cluster to the non-heme iron center (Fig. 2b, c)^10,40,41^.

**Fig. 3:**
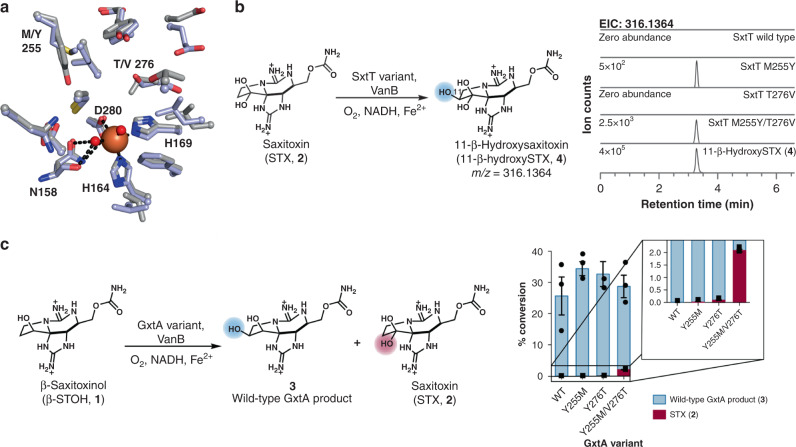
Biochemical analysis of SxtT and GxtA variants reveals changes in reaction selectivity. **a** The active sites of SxtT (purple) and GxtA (gray) are relatively similar to one another except for the replacement of SxtT residues Met255 and Thr276 with GxtA residues Tyr255 and Val276. **b** Reactions of SxtT wild-type and variants with STX (**2**). **c** Reactions of GxtA wild-type and variants with β-STOH (**1**) and the mixtures of products generated in these reactions. Data are presented as mean values ± SD. Reaction conditions: 5 µM SxtT/GxtA, 5 µM VanB, 200 µM β-STOH (**1**), 500 µM NADH, incubated at 30 °C for 2 h.

According to the DALI server^42^, SxtT and GxtA have the highest structural homology to dicamba monooxygenase (3GOB^35^) and KshA (4QDC^15^). Like these structures, SxtT and GxtA house the characteristic Rieske cluster motif and the 2-His, 1-Asp facial triad for coordinating the non-heme iron site^10^ (His164, His169, and Asp280). The octahedral iron coordination environment in SxtT and GxtA is completed by two water molecules, one of which is held in place through interactions with the backbone and sidechain of Asn158 (Fig. 3a). Although this Asn residue is conserved in many other structurally characterized α-only oxygenases^15,33–35,43^, its functional significance is unclear.

Notably, comparison of the SxtT and GxtA active site pockets reveals two differences, namely the inversion of a hydrophobic residue and a polar residue at positions 255 and 276. In SxtT, these positions correspond to Met and Thr residues, whereas in GxtA, they correlate with Tyr and Val residues, respectively (Fig. 3a). Importantly, these positions bracket the mononuclear iron site and are anticipated to play a role in positioning the substrate, dictating the selectivity of the catalyzed reactions (Fig. 3a).

### SxtT and GxtA mutagenesis results in added functionality

The roles of residues at the 255 and 276 positions in SxtT and GxtA were probed using site-directed mutagenesis to generate single and double variants of SxtT (M255Y, T276V, and M255Y/T276V) and GxtA (Y255M, V276T, and Y255M/V276T, Supplementary Tables 1 and 2). Each variant was expressed, purified, and analyzed by quantitative LC-MS in reactions using VanB as the electron donor (Fig. 3b, c and Supplementary Fig. 2)^16^. Each enzyme variant was assessed with β-STOH (**1**) and STX (**2**) to interrogate changes in the site-selectivity of the reactions.

Variants possessing a single substitution at the 276 position are biased toward wild-type site-selectivity on native substrates (i.e. SxtT with β-STOH (**1**) and GxtA with STX (**2**), Supplementary Figs. 6–8). The efficiency of the SxtT T276V variant was especially low due to precipitation upon the addition of substrate, however, a small amount of exclusively the native product STX (**2**) was formed. The GxtA V276T variant proved to be more stable under our reaction conditions, and we observed a small amount of dihydroxylated product formed in reaction with β-STOH (**1**), suggesting the variant is capable of hydroxylation at both the C11 and C12 positions (Supplementary Fig. 9). This ability in the GxtA V276T variant, which has two H-bond-donating residues in the active site, is mirrored by the corresponding SxtT M255Y variant. Reactions with the SxtT M255Y variant revealed a small amount of non-native hydroxylation activity on STX (**2**), a substrate which is not accepted by wild-type SxtT, generating 11-β-hydroxySTX (**4**, Supplementary Fig. 6). The corresponding GxtA variant, GxtA Y255M, was stable to standard reaction conditions with β-STOH (**1**), but did not demonstrate a change in selectivity, generating only **3**. Interestingly, both the SxtT and GxtA double variants displayed altered site-selectivity relative to the wild-type enzymes. In addition, SxtT M255Y/T276V demonstrated the ability to hydroxylate STX (**2**) more efficiently than SxtT M255Y. Analysis by LC-MS and MS/MS confirmed that SxtT M255Y and SxtT M255Y/T276V were capable of hydroxylating STX (**2**) at the C11 β-position, generating the native GxtA product, 11-β-hydroxySTX (**4**, Supplementary Fig. 6). The catalytic efficiencies of these reactions were evaluated using steady-state kinetics in comparison to the wild-type GxtA reaction with STX (**2**, Supplementary Figs. 10–12)^44^. The reaction of GxtA with STX (**2**) demonstrated an apparent catalytic efficiency (*k*_cat_/*K*_M_) of 6600 M^−1^ s^−1^. By contrast, the apparent *k*_cat_/*K*_M_ of SxtT M255Y with STX (**2**) was ~440 times lower at 15 M^−1^ s^−1^. However, introducing a second GxtA-like residue to generate SxtT M255Y/T276V revealed improvement in catalysis with STX (**2**) with an apparent *k*_cat_/*K*_M_ of 41 M^−1^ s^−1^.

Analysis of reactions between GxtA and its non-native substrate β-STOH (**1**) is complicated by co-elution of **3** and STX (**2**), the two potential products (Supplementary Fig. 7b). To differentiate **3** from STX (**2**) in reactions with GxtA variants, we leveraged the equilibrium exhibited by the hydrated ketone moiety of STX (**2**)^45^. At neutral pH, ethanol (EtOH) can be incorporated into STX (**2**) to form a hemiketal, enabling the unambiguous distinction between hydroxylation at C12 (EtOH incorporation) or C11 (no EtOH incorporation, Supplementary Figs. 13and 14) by LC-MS. Upon addition of EtOH to a reaction of GxtA Y255M/V276T with β-STOH (**1**), both an EtOH-incorporated product and a non-EtOH incorporated product were observed. This result suggests both STX (**2**) and **3** were generated (Fig. 3c and Supplementary Fig. 14), indicating that the GxtA double variant can perform reactions with both wild-type SxtT and GxtA activities. Comparing conversions of GxtA, GxtA Y255M, GxtA V276T, and GxtA Y255M/V276T with β-STOH (**1**) after 2 h revealed approximately equivalent total percent conversions for each enzyme (Fig. 3c, Supplementary Fig. 13 and 14, and Supplementary Table 4), suggesting that the overall level of catalysis is not impaired. Small quantities of STX (2) were generated as substitutions were introduced, making the GxtA active site more similar to SxtT, with the most C12 hydroxylation observed in GxtA Y255M/V276T (Fig. 3c). Together, these results indicate the two residues identified structurally alter C–H hydroxylation selectivity, resulting in gain of function variants.

### Substrate analog bound structures reveal an essential hydrogen bond

**Fig. 4:**
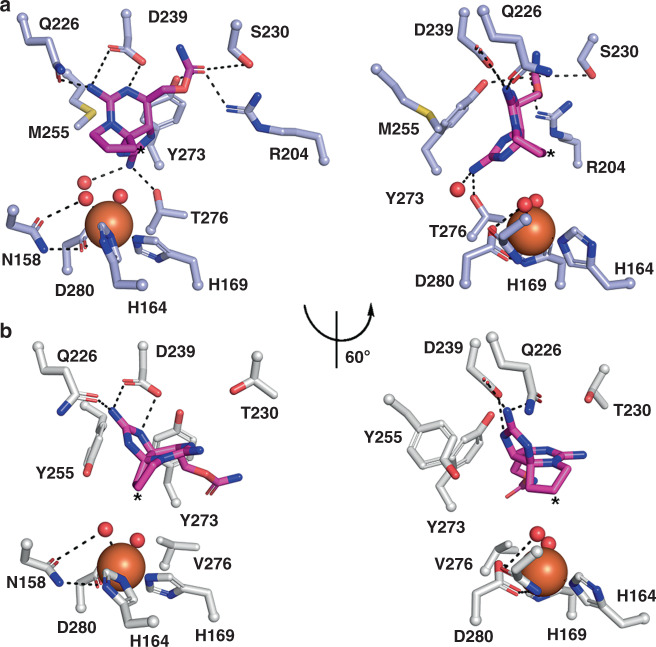
Structures of SxtT and GxtA with ddSTX bound reveal the protein interactions that are important for correctly positioning substrate for activation. **a** ddSTX (dark pink sticks) is anchored in the active site of SxtT via interaction with several different residues that are highlighted here. A rotated view of the active site reveals that C12 (asterisk) is the closest position of ddSTX to the non-heme iron site. **b** The positioning of ddSTX in the GxtA active site places C11 (asterisk) closest to the iron center. Relative to the placement of ddSTX in SxtT, the guanidinium group that interacts with Thr276 rotates nearly 120º to istead interact with Gln226 and Asp239 in the GxtA active site. Both of these residues are conserved in SxtT.

To further probe the selectivity differences in SxtT and GxtA, the structures of each protein bound to dideoxysaxitoxin (ddSTX) were determined (Supplementary Table 3). ddSTX has previously been shown to serve as a substrate for both SxtT and GxtA and exhibits reduced toxicity relative to STX and β-STOH^16^. This lowered toxicity, coupled with the availability of the molecule through synthesis, rendered ddSTX a prime target for the pursuit of substrate-bound structures. The ddSTX-bound structures are relatively similar to those of the native enzymes (r.m.s.d determined by PyMol of 0.338 and 0.491 Å over 1002 and 952 Cα atoms for SxtT and GxtA, respectively) except for the presence of one ddSTX molecule in the active site of each monomer (Supplementary Figs. 3–5). The binding of ddSTX in the active site of SxtT involves hydrogen bonds between the carbamate sidechain and Ser230, Tyr273, and Arg204, as well as interactions between the guanidinium group of the six-membered ring with Asp239 and Gln226 (Fig. 4a). The ddSTX molecule is also held in the active site by two interactions with the five-membered cyclic guanidinium group of ddSTX, which participates in hydrogen bonds with Thr276 and a water molecule that bridges ddSTX to the non-heme iron ligand Asp280 (Fig. 4a, Supplementary Fig. 4a, and Supplementary Fig. 5a, c, d). Along with Thr276, each formed interaction anchors ddSTX such that C12 is the closest atom of the molecule to the non-heme iron site and positions the α-C–H bond for hydrogen atom abstraction (Fig. 4a and Supplementary Fig. 4a). Met255 appears to be important for spatially directing the binding of ddSTX in the SxtT active site as it packs against the six-membered guanidinium ion ring. Importantly, as residues Tyr273, Asp239, and Gln226 are conserved in GxtA and Arg204 and Ser230 are substituted with Lys and Thr residues (Supplementary Fig. 1), correct placement of the C12 α-C–H bond for hydroxylation appears to be largely dictated by the bulk and hydrophobicity of Met255 and the hydrogen bond with Thr276. Indeed, the replacement of these two residues in the GxtA active site with Tyr and Val residues results in significant movement of ddSTX such that it interacts only with conserved residues, Asp239 and Gln226 (Fig. 4b, Supplementary Figs. 4b and 5b, e, f).

### Identification of a putative active site tunnel in SxtT and GxtA

**Fig. 5:**
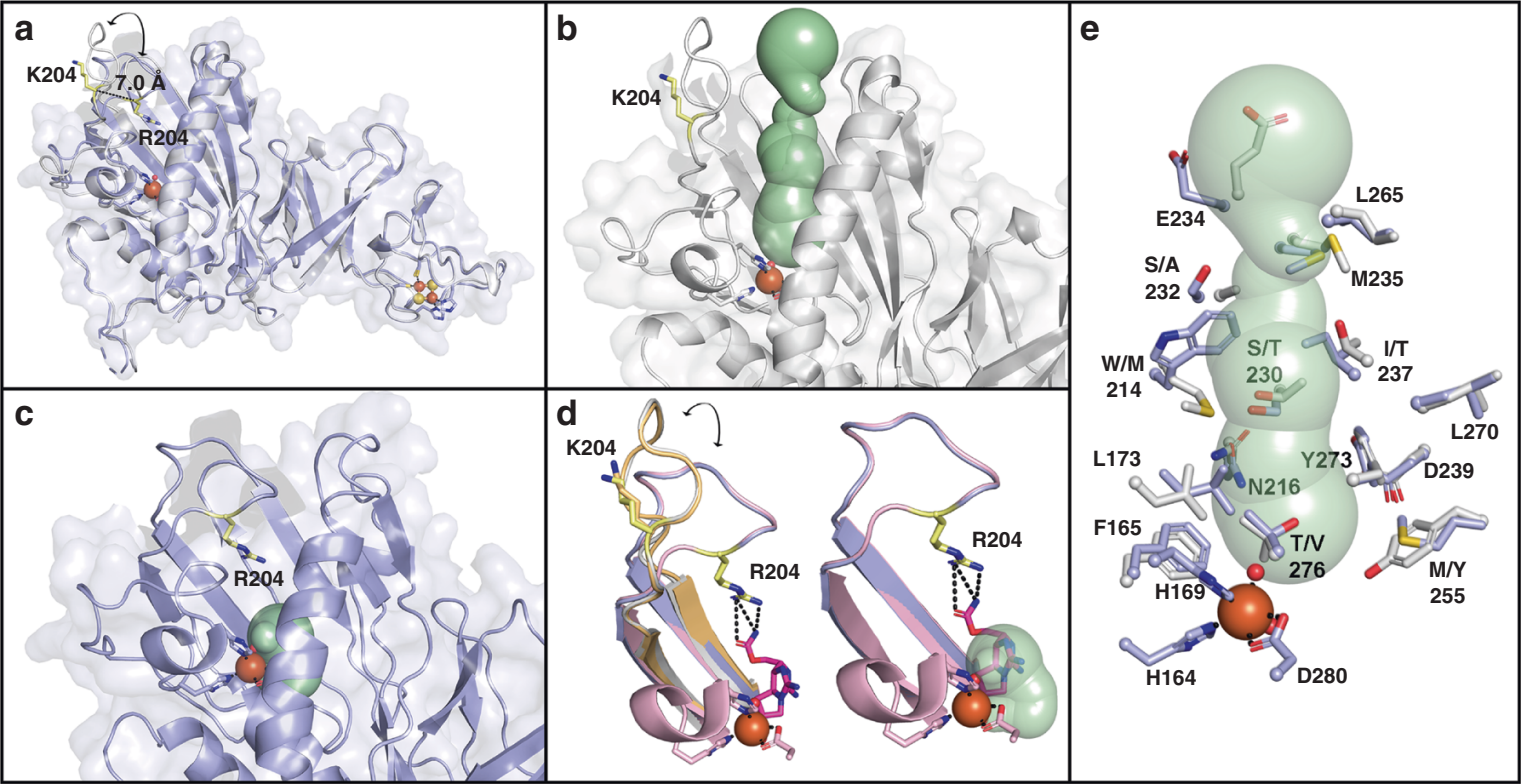
SxtT and GxtA exhibit different conformations of a loop that appears to gate access to the active site. **a** An overlay of the SxtT and GxtA monomers reveals a 7.0 Å difference in the location of the residue at the 204 position. This difference is due to changes in the position of a loop that spans the range 195–215. **b** The open conformation of the loop in GxtA allows visualization of a tunnel that leads directly from the protein surface to the active site. **c** The closed orientation of the loop in SxtT restricts calculation of an equivalent tunnel, but instead allows visualization of the active site cavity. **d** An overlay of all four structures determined in this work reveals that Arg204 in the co-crystal structure assumes a similar position to that seen in the ligand free structure of SxtT. In this orientation, Arg204 interacts with ddSTX. **e** An overlay of SxtT and GxtA shows differences in the residues that line the putative tunnel to the active site. In all panels, structures are shown for SxtT, GxtA, ddSTX-SxtT, and ddSTX-GxtA in purple, gray, pink, and orange respectively. Tunnels are colored mint, ddSTX is dark pink, and the Arg/Lys204 residue is yellow.

The double variant enzymes unexpectedly have the ability to perform additional reactions with the selectivity of the opposite enzyme, but remain biased toward the site-selectivity of the native reaction, as evidenced by the GxtA reaction with β-STOH (**1**). Thus, we decided to investigate differences in residues located outside of the active sites. We found that each of the structures determined in this work possess subunits that are missing some residues in a loop that spans the range 195–215. These residues are too disordered to be confidently built into the refined structures. However, in one subunit of both the ligand-free SxtT and GxtA structures, the loop is ordered and can be visualized. Comparison of these ordered subunits shows that this loop assumes remarkably different conformations. In SxtT, this loop folds in and reaches toward the active site pocket, whereas in GxtA, this loop adopts an extended conformation on the surface of the protein (Fig. 5a). These loop conformations were further investigated using the MOLEonline server^46,47^ to calculate tunnels present in our structures. Through this analysis, we were able to visualize a tunnel in GxtA that leads from the surface of the protein to the active site (Fig. 5b). An equivalent tunnel in SxtT does not exist due to the orientation of the loop, which closes off the active site cavity (Fig. 5c). We likely benefitted from the crystal contacts in capturing two states of the loop orientation, but based on the location of the active site and the flexibility of this loop in all of the other subunits, we hypothesize that this loop samples a multitude of orientations that either restrict or permit access to the active site. Consistent with this loop serving as a gate to the active site, in our ddSTX-bound SxtT structure, this loop partially orders in each subunit and interacts with the bound ddSTX molecule, which is found at the base of the calculated tunnels (Fig. 5d). Closer inspection of the tunnel also revealed differences in the tunnel-lining residues, which may be important for guiding the substrate into its position in the active site (Fig. 5e).

## Discussion

Biocatalysts for C–H hydroxylation are underexploited in chemical synthesis due to our limited understanding of structure-function relationships. This is particularly evident in the Rieske oxygenase family of enzymes, which are structurally underexplored. Here, we examined two biosynthetic Rieske monooxygenases, SxtT and GxtA, that catalyze site- and stereoselective reactions in PST biosynthesis. By solving structures of SxtT and GxtA, we identified two divergent residues in the active sites of these enzymes that bracket the non-heme iron site. We tested the importance of these residues in dictating the site-selectivity of the catalyzed reactions by replacing one or two residues in each protein and gauging the ability of these enzymes to catalyze hydroxylation at either C11 or C12 (Fig. 3). We found that the removal of a hydrogen bond donor from the active site to generate SxtT T276V resulted in precipitation under our reaction conditions with every substrate. The removal of a hydrogen bond donor to generate GxtA Y255M resulted in trace amounts of dihydroxylation activity. In contrast, the addition of a hydrogen bond donor to the active site of both enzymes (SxtT M255Y and GxtA V276T) permits a small amount of monohydroxylation to occur at the non-native site. For each double variant we saw that the amount of hydroxylation at these non-native positions increased.

We further revealed, by solving ddSTX-bound structures of SxtT and GxtA, that these residues are important for positioning substrate in the active site. In SxtT, Thr276 hydrogen bonds to the five-membered ring containing a guanidinium ion and Met255 sterically orients ddSTX for catalysis at C12 (Fig. 4a). In GxtA, rotation about the carbamate group instead positions ddSTX for hydrogen-atom abstraction at C11 (Fig. 4b). Intriguingly, each of the observed interactions between ddSTX and GxtA are with residues that are conserved in SxtT, highlighting the importance of Thr276 for correctly positioning C12 for hydroxylation. However, it has not escaped our notice that the addition of a hydroxyl group to the C12 α-position of ddSTX (identical to that found in the native GxtA substrate) would provide an additional interaction with Tyr255 in the GxtA active site, further supporting our data and the proposed importance of this residue for selectivity (Fig. 4b, Supplementary Figs. 4b, and Supplementary Fig. 5b, e, f).

Surprisingly, the double variant enzymes remain biased toward native reaction site- and stereoselectivity. This incomplete switch in selectivity could be due to distinct conformational changes upon substrate binding as well as sequence differences in the composition of the flexible loop or substrate tunnel identified in these enzymes (Fig. 5). Indeed, although we find that these enzymes share 88-percent sequence identity with one another, the 195–215 loop is only 52-percent identical in sequence and only 29% identical over the portion (201–214) that plugs the active site tunnel in SxtT. Different orientations of the equivalent loop have been observed in dicamba monooxygenase^48^ and NdmA/NdmB^37^ and, similar to what we propose for SxtT and GxtA, have been suggested to either permit or restrict access of substrate to the active site and/or promote product release. Consistent with this hypothesis, GxtA (which showcases the “open” conformation) exhibits a broader substrate scope than SxtT^16^. We also find that the residues lining the putative active site tunnel vary between SxtT and GxtA; several residues in SxtT (Trp314, Ile237, and Ser232) that are larger than their counterparts in GxtA (Met214, Thr237, and Ala232) are found near the entrance to the active site. These differences may bias substrate positioning, serve a role in substrate recognition, or gate substrate access to the active site. In agreement with this hypothesis, recent computational studies on naphthalene dioxygenase predict that residues in an equivalent region may also play roles in controlling substrate access to the active site^49^. Finally, it is also a possibility that the protein forms interactions with hydroxyl groups present on the native substrates of each enzyme that are not observed with ddSTX. Further studies to determine the importance of non-conserved residues in the tunnel and loop region and obtain crystal structures with native substrates bound in the active site of each enzyme will be pursued to further define the mechanisms of selectivity in SxtT and GxtA.

Understanding the basis for divergent selectivity has implications for engineering these enzymes as biocatalysts for the generation of nonnative PST analogs for pharmacological studies. Although there is a still much to learn about the Rieske oxygenase superfamily, the structural characterization of SxtT and GxtA provides information on this understudied class of non-heme monooxygenases and reveals several architectural components that are important for the selectivity of these enzymes.

## Methods

### Site-directed mutagenesis

Touchdown PCR^50^ was used to introduce mutations and amplify sequences. In all, 25 µL reactions composed of 1x Phusion buffer (HF), 200 µM dNTPs, 2 ng/µL plasmid template (His-SxtT-pET151 and His-GxtA-pMCSG7), 1 µM primer (Supplementary Table 2), 1 U Phusion DNA polymerase and 1% DMSO. Reactions were run using the following program: 98 °C 30 s, (98 °C 10 s, 69–63 °C 30 s decreasing in 0.5 °C increments each cycle) and 72 °C 7 min) for 12 cycles followed by (98 °C 10 s, 63 °C 30 s, 72 °C 7 min) for 22 cycles, and 72 °C for 15 min. Digestion with DpnI was performed by combining 8 µL PCR reaction with 1 µL 10x CutSmart buffer and 1 µL DpnI (20,000 U/mL stock). The DpnI digest reaction was incubated at 37 °C for 3 h and the 10 µL reaction volume was transformed into DH5α chemically competent cells. Mutations were verified by Sanger sequencing (University of Michigan).

### Expression protocol for SxtT and GxtA wild-type and variants

For protein to be prepared for crystallography, pMCSG9 plasmids containing *sxtT* or *gxtA* were transformed by standard heat-shock protocols into chemically competent C41(DE3) *E. coli* cells to generate MBP-SxtT and MBP-GxtA. For protein to be prepared for reactions, a pET151 vector containing *sxtT* and a pMCSG7 vector containing *gxtA* were used to generate His-SxtT and His-GxtA. The remaining expression procedures are identical for both sets of constructs. A single colony was used to inoculate 10 mL of LB containing 100 µg/mL ampicillin and incubated overnight at 37 °C, 200 rpm overnight. The overnight culture was used to inoculate a 1 L LB culture in a 2.8 L flask containing 100 µg/mL ampicillin. Cultures were grown at 37 °C and 200 rpm until the OD_600_ reached 0.6–0.8. Flasks were briefly cooled to 20 °C before addition of 0.1 mM IPTG, 0.2 mg/mL ferric ammonium citrate, and 0.4 mg/mL ferrous sulfate heptahydrate. Cultures were incubated at 20 °C and 200 rpm for ~18 h before harvesting. The average pellet for a 1 L culture was 5 g wet cell mass.

### Expression protocol for VanB

pMCSG7 plasmid containing *vanB* was transformed by heat-shock into chemically competent BL21(DE3) *E. coli* cells^16^. A single colony was used to inoculate 10 mL LB containing 100 µg/mL ampicillin and incubated at 37 °C, 200  rpm overnight. The overnight culture was used to inoculate a 1 L LB culture in a 2.8 L flask containing 100 µg/mL ampicillin. Cultures were incubated at 37 °C and 200 rpm until an OD_600_ of 0.6–0.8 was achieved. Flasks were chilled at 20 °C for 1 h before induction by addition of 0.1 mM IPTG. Cultures were incubated for ~18 h before harvesting. The typical wet mass of a pellet from a 1 L culture was 6 g.

### Purification protocol for SxtT and GxtA for reactions

Cell pellet from 6 L culture of His-SxtT or His-GxtA expressions (~30 g) was resuspended in 120 mL lysis buffer (50 mM Tris-HCl pH 7.4, 300 mM NaCl, 10% glycerol, 10 mM imidazole). Cells were lysed by sonication, 5 min total “on” time, 10  on, 20 s off. Lysed cells were centrifuged at 40,000 × *g* for 30 min and the supernatant was filtered using 0.45 µM syringe filters (CellTreat). The sample was loaded onto an ÄKTA Pure FPLC system fitted with a 5 mL HisTrap column, where Buffer A was the lysis buffer and Buffer B was the lysis buffer with the addition of 400 mM imidazole. Lysate was loaded onto the column at 2.5 mL/min, washed with 6 CV of 40 mM imidazole buffer at 2.5 mL/min, and eluted in a 15 CV gradient to 100% Buffer B at 1 mL/min. In all, 2 mL fractions were collected during elution. The fractions containing desired protein were pooled and concentrated to 2 mL using a 30  kDa cutoff centrifugal filter and diluted to 50 mL with anion exchange binding buffer (20 mM Tris-HCl pH 7.0, 50 mM NaCl). The diluted mixture was loaded onto a 5 mL HiTrap Q HP column at 2.5 mL/min and eluted in an 8 CV gradient to 100% anion exchange elution buffer (20 mM Tris-HCl pH 7.0, 1 M NaCl). The fractions containing desired protein were pooled and diluted to 30 mL with TEV dialysis buffer (20 mM Tris-HCl pH 7.4, 50 mM NaCl, 1 mM DTT, 10% glycerol). In all, 2 mg of TEV protease was added to the diluted protein and the mixture was dialyzed overnight. The next day, the contents of the dialysis bag were combined with 1 mL Ni-NTA resin and incubated for 1 h with rocking. The mixture was poured onto a 12 mL column and allowed to drain completely. The flowthrough containing His-cleaved protein was collected and concentrated to at least 100 μM and flash frozen in liquid nitrogen for long-term storage at −80 °C.

### Purification protocol for SxtT and GxtA for crystallography

Cell pellet from 6 L culture of MBP-SxtT or MBP-GxtA expressions (~30 g) was resuspended in 120 mL lysis buffer (20 mM Tris-HCl pH 7.0, 1 M NaCl, 1 mM DTT). Cells were lysed by sonication, 5 min total “on” time, 10 s on, 20 s off. Lysed cells were centrifuged at 40,000×*g* for 30 min and the supernatant was filtered using 0.45 µM syringe filters. The sample was loaded onto an ÄKTA Pure FPLC system fitted with a 5 mL MBPTrap column, where Buffer A was the lysis buffer and Buffer B was the lysis buffer with the addition of 10 mM maltose. Lysate was loaded onto the column at 2.5 mL/min, washed with 10 CV of lysis buffer at 2.5 mL/min, and eluted in a 5 CV gradient to 100% Buffer B at 1 mL/min. Fractions containing desired protein were pooled and diluted to 30 mL.In all, 2 mg of TEV protease was added to the diluted protein and the mixture was dialyzed overnight in 20 mM Tris-HCl pH 7.4, 50 mM NaCl, 1 mM DTT, 10% glycerol buffer. The next day, the contents of the dialysis bag were combined with 1 mL Ni-NTA resin that had been washed with water and incubated for 1 h with rocking. The mixture was poured over a 12 mL column and allowed to drain completely. The flowthrough containing proteins cleaved from MBP were collected and concentrated to 2 mL using a 30 kDa cutoff centrifugal filter. The concentrated protein was diluted to 50 mL in anion exchange binding buffer (20 mM Tris-HCl pH 7.0, 50 mM NaCl). The diluted mixture was loaded onto a 5 mL HiTrap Q HP column at 2.5 mL/min and eluted in an 8 CV gradient to 100% anion exchange elution buffer (20 mM Tris-HCl pH 7.0, 1 M NaCl). The fractions containing desired protein were pooled, concentrated to 2 mL, and injected onto a Sephacryl S-200 HR gel filtration column equilibrated with 20 mM Tris-HCl pH 7.4, 200 mM NaCl. Desired protein was pooled and exchanged into 50 mM HEPES pH 8.0, 10% glycerol buffer using a PD-10 desalting column following the manufacturer’s protocol. Desalted protein was concentrated to a minimum of 10 mg/mL.

### Purification protocol for VanB

Cell pellet was resuspended in 4 mL of lysis buffer (50 mM Tris-HCl pH 7.4, 300 mM NaCl, 10 mM imidazole, 10% glycerol) per gram of pellet^16^. The mixture was sonicated for 5 min total “on” time, 10 s on, 20 s off. Lysed cells were centrifuged at 40,000×g for 30 min and the clarified supernatant was combined with 4 mL Ni-NTA resin. The mixture was incubated for 2 h and poured over a 35 mL column. The packed resin was washed with 10 mL lysis buffer followed by 10  mL each of buffer containing increasing amounts of imidazole: 25, 30, and 35 mM imidazole. Proteins were eluted with 20 mL elution buffer (50 mM Tris-HCl pH 7.4, 300 mM NaCl, 250 mM imidazole, 10% glycerol). Fractions containing protein were run on an SDS-PAGE gel and those containing VanB were pooled and exchanged into storage buffer (50 mM Tris-HCl pH 7.4, 10% glycerol) using a PD-10 desalting column. Proteins were concentrated to 206 μM and flash frozen in liquid nitrogen for long-term storage at −80 °C.

### Crystallization of SxtT, ddSTX-bound SxtT, GxtA, and ddSTX-bound GxtA

The conditions for crystallizing SxtT were identified anaerobically using a Mosquito pipetting robot (TTP LabTech) that is housed in a Coy chamber (Coy Lab Products) maintained using a 95% nitrogen and 5% hydrogen mixture at room temperature. Initial crystals of SxtT grew within one week of mixing 0.3 µL of 10 mg/mL SxtT (50 mL HEPES pH 8.0, 10% v/v glycerol) with 0.3 µL of crystallization solution (2.0 M (NH_4_)_2_SO_4_, 0.1 M Bis-Tris pH 6.5). The crystals were subsequently optimized by mixing SxtT in 1:1 proportion with a well solution of (2.0 M (NH_4_)_2_SO_4_, 0.1 M Bis-Tris pH 6.5, 10% v/v glycerol) in a hanging drop vapor-diffusion experiment. Brown crystals appeared in three days at 20 °C and achieved maximum size over the course of two weeks. Crystals of ddSTX-bound SxtT were grown similarly except the protein concentration was adjusted to 8 mg/mL and the protein buffer was altered to include 20 mM ddSTX. GxtA crystal conditions were identified similarly and grew within three days of mixing 0.3 µL of 10 mg/mL GxtA (50 mL HEPES, 10% v/v glycerol pH 8.0) with 0.3 µL of crystallization solution (0.2 M MgCl_2_, 0.1 M Bis-Tris pH 5.5, 25% v/v PEG3350). GxtA crystals were manually optimized using hanging drop vapor diffusion by changing the concentration of the protein (24 mg/mL) and changing the well solution (0.3 M MgCl_2_, 0.1 M Bis-Tris pH 5.5, 25% v/v PEG3350, 15% v/v glycerol). Crystals of ddSTX-bound GxtA were grown similarly except the protein concentration was adjusted to 19.2 mg/mL and the protein buffer was altered to include 20 mM ddSTX. Brown crystals appeared in 1 day following incubation at 20 °C and achieved maximum size within 1 week. All crystals were harvested and cryocooled in the coy chamber with no additional cryoprotectant added.

### Data processing and structure solution

The datasets for SxtT, GxtA, ddSTX-bound SxtT, and ddSTX-bound GxtA were collected at the Life Sciences Collaborative Access Team beamlines 21-ID-G (SxtT, Rayonix MX 300), 21-1D-F (ddSTX-bound GxtA, Rayonix MX 300), and 21-ID-D (GxtA and ddSTX-bound SxtT, Dectris Eiger 9M) at the Advanced Photon Source, Argonne National Laboratory. All datasets were collected at a temperature of 100 K and wavelengths of 0.97856 (SxtT), 0.97872 (ddSTX-bound GxtA) and 1.1272 Å (GxtA and ddSTX-bound SxtT). Indexing, integration, and scaling of the data were performed in HKL2000^51^. SxtT and ddSTX-bound SxtT indexed as *C*222, whereas GxtA and ddSTX-bound GxtA indexed as *P1*2_1_1. Both structures have three molecules in the asymmetric unit. The structure of SxtT was solved using an edited model of dicamba monooxygenase (31% identity, 48% similarity)^35^ for molecular replacement. This modified model lacked the Rieske cluster, non-heme iron site, water molecules, and had the sidechains pruned to the most common atom using Phenix Sculptor^52^. With this modified model, a molecular replacement solution (LLG score of 69 and Z score of 10.8) was determined using Phenix AutoMR^53^. The resulting structure of SxtT, in the absence of water molecules and cofactors was used to subsequently determine the structures of ddSTX-bound SxtT by isomorphous replacement and GxtA by molecular replacement (LLG = 8968, *Z* score = 85.4). Once complete, the structure of GxtA was used to determine the structure of ddSTX-bound GxtA. For all structures described in this work, model building, model adjustment, the building of metal sites, and the addition of water molecules were performed in COOT^54^. Iterative rounds of structure and B-factor refinement were performed in Phenix^53^. An initial five cycles of simulated annealing were used to reduce model bias. Identical *R*_free_ test sets composed of five-percent of the original data were used for native and ddSTX-bound structures.

Small molecule parameter files were generated using the chemical SMILES string and the electronic Ligand Builder and Optimization workbench, eLBOW^55^, in Phenix for ddSTX. In parallel, small molecule parameter files were generated using the Grade Web Server Global Phasing, Cambridge, UK) for comparison. Although both methods resulted in similar placement of the ligand in the electron density, the latter method resulted in more accurate refined geometry. Thus, the latter was used in refinements for both ddSTX-bound structures determined in this work. For SxtT, ddSTX is modeled at full occupancy in chains A and C, whereas it is modeled at 85% occupancy in chain B. For GxtA, ddSTX is modeled at full occupancy in all three chains.

The final structures were analyzed using simulated annealing composite omit electron density maps. The final SxtT structure was further scrutinized using the MolProbity program^56^. This structure is missing residues in each monomeric subunit (A:1, 205-208, and 297-303, B: 298-303, and C: 201-212, and 298-303) and has 96.3-, 3.7-, and 0-percent of residues in the favored, allowed, and disallowed regions of the Ramachandran plot, respectively. Similarly, for ddSTX-bound SxtT, which is also missing residues in each monomer (A:1, 297-303, B: 1, 297-303, and C: 1, 297-303, and 204-213), there are also 96-, 4-, and 0-percent of residues are in the favored, allowed, and disallowed regions of the Ramachandran plot, respectively. The GxtA and ddSTX-bound GxtA structures are also missing residues in each subunit. For GxtA, these resides are: A: 1, 177-178, 297-303, and 201-213, B: 1, 204-208, 298-304, and C: 1 and 296-303. This structure has 96.5%, 3.5%, and 0% of residues in the favored, allowed, and disallowed regions of the Ramachandran plot, respectively. For the ddSTX-bound GxtA structure, which has 96.7%, 3.3%, and 0% of residues in the favored, allowed, and disallowed regions of the Ramachandran plot, respectively, the missing residues are A: 1, 296-305, and 202-213, B: 1, 204-209, 298-304, and C: 1 and 296-303. Data statistics are summarized in Supplementary Table 3. All structure figures were made using PyMol and the crystallography software packages were compiled by SBGrid^57^.

### Calculation of active site tunnels in SxtT and GxtA

The existence of an active site tunnel in SxtT and GxtA was analyzed using the MOLEonline server^46,47^. In brief, the monomeric subunits which contained an ordered 195–215 loop was loaded into the server. The channel parameter settings were adjusted to tunnels that originate from the same starting point and detect pores in all cavities was turned on and the non-heme iron site and ligands were manually input as a starting point for the analysis. Using these parameters, the tunnel from the surface of GxtA to the active site and the active site cavity in SxtT were found. The tunnels and tunnel-defining residues (entrance, exit, and bottleneck) were visualized using PyMol.

### LC-MS and MS/MS analysis

Liquid chromatography-mass spectrometry (LC-MS) analysis was performed on an Agilent G6545A quadrupole-time of flight (QTOF) or Agilent 6230 time of flight (TOF) mass spectrometer equipped with a dual AJS ESI source and an Agilent 1290 Infinity series diode array detector, autosampler, and binary pump. Solvent A = water with 0.1% formic acid. Solvent B = 95% acetonitrile, 5% water and 0.1% formic acid. An Acquity UPLE BEH Amide 1.7 µm, 2.1 × 100 mm hydrophobic interaction liquid chromatography (HILIC) column from Waters was used for all separations. The chromatographic method was typically 18% A 0–5  min at 0.4 mL/min or 15% A 0–7 min at 0.3 mL/min. In all, 0.3–10 µL injections were made for each sample. Targeted MS/MS was performed using the QTOF using an 18% A isocratic method, 0–5 min, to obtain fragmentation patterns. Methods were augmented to target each mass. Collision energies were set to 10, 20, and 30 eV and the resulting chromatograms were averages of the three collision energies.

### SxtT and GxtA wild-type and variants reactions

Stock solutions of all substrates and product standards were prepared to final concentrations of 20 mM in dimethyl sulfoxide (DMSO, analytical grade). Enzyme aliquots were discarded after one freeze-thaw cycle. Stocks of 1 mM Fe(NH_4_)_2_(SO_4_)_2_•6H_2_O were prepared fresh in MilliQ water before each use. In all, 10 mM stock solutions of nicotinamide adenine dinucleotide hydride (NADH) were prepared in water and stored at −20 °C, undergoing no more than 10 freeze-thaw cycles. All reactions were conducted in 1.5 mL plastic tubes and 96-well plates. In all, 50 µL reactions consisting of 5–20 µM SxtT or GxtA variant, 5  µM VanB, 200 µM β-STOH (**1**) or STX (**2**), 500 µM NADH, 100 µM Fe(NH_4_)_2_(SO_4_)_2_, and 50 mM Tris-HCl pH 7.0 buffer were combined and incubated at 30 °C for 2 h and quenched by the addition of 150 µL acetonitrile. Reactions were centrifuged at 12,000×*g* for 20 min to pellet precipitated material and 100 µL of the supernatant was diluted with sterile filtered acetonitrile containing 1% formic acid and 0.05% ^15^N-arginine as an internal standard for mass spectrometry analysis.

### Ethanol incorporation into reaction products

Reactions were performed as described above. After quenching with 150 µL acetonitrile and subsequent centrifugation, reactions were diluted 1:1 with sterile filtered acetonitrile containing 1% formic acid and 0.05% ^15^N-arginine as an internal standard. In all, 50 µL 100% ethanol was added to 50 µL of the reaction mixture containing internal standard and incubated at room temperature for at least 6 h prior to analysis by LC-MS. Quantification of EtOH-STX (**6**) in samples was based on the percent EtOH-STX (**6**) present in samples of STX (**2**) incubated with ethanol prepared on the same day, under the same conditions as the reactions to be analyzed. A standard curve of STX (**2**) was used to quantify the amount of STX (**2**) remaining in the samples treated with ethanol (Supplementary Fig. 12). The dilution factor was adjusted in the standard curve by adding additional acetonitrile in place of ethanol. The difference in ionization in a sample containing acetonitrile and a sample containing a mixture of acetonitrile and ethanol is anticipated to be negligible.

### Steady-state kinetics

To determine the steady-state kinetic parameters of GxtA, SxtT M255Y, and SxtT M255Y/T276V with STX (**2**), reactions were conducted on 50 μL scale in duplicate with substrate ranging 1–200 μM in duplicate with 5 μM enzyme, 5  μM VanB, 100 μM Fe_2_(NH_4_)_2_(SO_4_)_2_, and 50 mM Tris HCl pH 7 buffer in a 96-well plate. Reactions were initiated by the addition of 500 μM NADH (10 μL distributed by multichannel pipette). For GxtA and SxtT M255Y/T276V, reactions were quenched after 30 s by the addition of 10 μL of 10% formic acid in acetonitrile. 140 μL dilution mix containing internal standard was then added. 96-well plates were centrifuged at 2000 × *g* for 2 min, then 100 μL of the centrifuged mixture was added to a clean 96-well 0.22 μm filter plate. The plate was centrifuged over a clean 96-well plate at 2000 × *g* for 2 min. Product standards were prepared in the same manner with enzyme storage buffer (20 mM Tris HCl pH 7.4, 200 mM NaCl) replacing enzymes. In all, 5 μL each sample was injected on the TOF LC-MS. The resulting Michaelis–Menten curves for each substrate are shown in Supplementary Figs. 9–11 alongside standard curves used to quantify 11-β-hydroxySTX (**4**) product.

For SxtT M255Y, protocol adjustments were necessary to observe the low quantities of product being generated. The reactions were performed in quadruplicate and the most consistent three data points were used to generate the curve in Supplementary Fig. 11c. Reactions were quenched after 1 min by the addition of 10 μL of 10% formic acid in acetonitrile followed by the addition of 50 μL acetonitrile to precipitate protein. Ninety-six-well plates were centrifuged at 2000 × *g* for 2 min, then 50 μL each sample was combined with 10 μL dilution mix containing internal standard and added to a clean 96-well 0.22 μm filter plate. The plate was centrifuged over a clean 96-well plate at 2000 × *g* for 2 min. In all, 10 μL each sample was injected on the QTOF LC-MS for analysis. Data was collected in Microsoft Excel and analyzed using Graphpad Prism.

### Quantification of GxtA reaction products with β-STOH as a substrate

Triplicate reactions consisting of 5 µM GxtA, 5 µM VanB, 500 µM β-STOH (**1**), 500 µM NADH, 100 µM Fe(NH_4_)_2_(SO_4_)_2_ and 50 mM Tris HCl pH 7.0 buffer were incubated at 30 °C for 2 h. Reactions were quenched and incubated with ethanol as described above. The quantity of β-STOH (**1**) remaining in the reactions and the quantity EtOH-STX (**6**) present were determined using standard curves of β-STOH (**1**) (Supplementary Fig. 13b) and EtOH-STX (**6**) (Supplementary Fig. 13c). The quantity **3** generated in each reaction was determined by subtracting the determined quantities of β-STOH (**1**) and EtOH-STX (**6**) from 500 µM (the amount of β-STOH (**1**) added to the reaction).

### Synthesis and standard preparation

Product standards of ddSTX, β-STOH (**1**), STX (**2**), and 11-β-hydroxySTX (**4**) were prepared as previously described^16^. Briefly, STX (**2**) was extracted from *Alexandrium* cultures and purified. β-STOH (**1**) was prepared from STX (**2**) by reduction with sodium borohydride. 11-β-hydroxySTX (**4**) was prepared through hydrolysis of the sulfated PST analog gonyautoxin 3 that had been extracted from *Alexandrium* cultures. ddSTX was synthesized according to procedures described by Mulcahy and coworkers^58^.

## Supplementary information

Supplementary Information

Reporting Summary

## Data Availability

Protein coordinates and structure factors have been submitted to the Protein Data Bank under accession codes 6WN3 (SxtT), 6WNC (GxtA), 6WNB (ddSTX-bound SxtT), and 6WND (ddSTX-bound GxtA). The source data underlying Fig. 3c and Supplementary Figs. 10-14 are provided as a Source data file. Other data are available in the Supplementary Information and from the corresponding authors upon reasonable request.

## Source Data

Source Data
