# Supplementary material for: Structural basis for divergent C–H hydroxylation selectivity in two Rieske oxygenases
Source: Nat Commun. 2020 Jun 12;11:2991. doi: 10.1038/s41467-020-16729-0 (PMC7293229; doi:10.1038/s41467-020-16729-0)
Supplement: Supplementary file 1 — Supplementary Information [file 41467_2020_16729_MOESM1_ESM.pdf]

Supplementary Information

Structural basis for divergent C–H hydroxylation selectivity in two Rieske oxygenases

April L. Lukowski,[‡] Jianxin Liu[‡] Jennifer Bridwell-Rabb and Alison R. H. Narayan

[‡]These authors contributed equally to this work.

Table of Contents

Supplementary Figure 1. Alignment of SxtT an GxtA protein sequences 3
Supplementary Figure 2. SxtT and GxtA variants protein gel 4
Supplementary Figure 3. ddSTX bound in SxtT and GxtA........ 5
Supplementary Figure 4. Stereo image of ddSTX bound in SxtT and GxtA 6
Supplementary Figure 5. 2Fo-Fc and Fo-Fc maps of ddSTX-bound structures 7
Supplementary Figure 6. Reaction of STX with WT GxtA and SxtT variants........ 9
Supplementary Figure 7. LC-MS analysis of reactions of active site variants 10
Supplementary Figure 8. MS/MS spectra of active site variant reaction products........ 11
Supplementary Figure 9. Dihydroxylation by GxtA V276T and Y255M/V276T........ 12
Supplementary Figure 10. GxtA steady-state kinetic analysis with STX........ 12
Supplementary Figure 11. SxtT M255Y/T276V steady-state kinetic analysis with STX 12
Supplementary Figure 12. SxtT M255Y steady-state kinetic analysis with STX........ 13
Supplementary Figure 13. Extent of ethanol incorporation into STX 13
Supplementary Figure 14. GxtA Y255M/V276T reaction scheme and standard curves........ 14
Supplementary Table 1. Protein sequence acccession numbers........ 15
Supplementary Table 2. Site-directed mutagenesis primers........ 15
Supplementary Table 3. Data collection and refinement statistics 16
Supplementary Table 4. Summary of GxtA EtOH-incorporation results with β-STOH 17

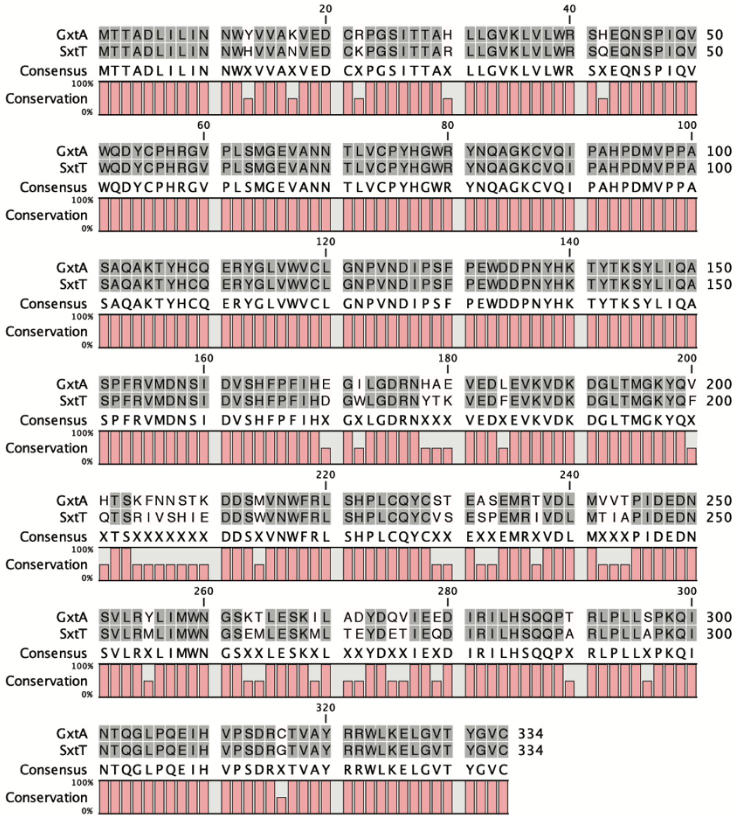

Supplementary Figure 1. Alignment of SxtT and GxtA protein sequences reveals that these proteins share 88-percent sequence identity with each other. Conserved residues are shown in grey and divergent residues are not highlighted. Alignment generated using ClustalW and visualized by CLC Sequence Viewer (v7.6.1).

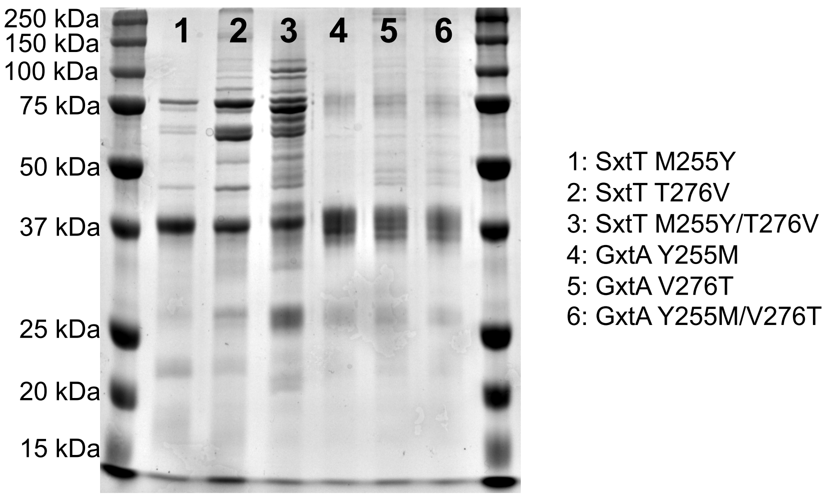

Supplementary Figure 2. SxtT and GxtA variants were expressed, purified by Ni-affinity chromatography, and used in enzymatic assays. Shown is an SDS-PAGE gel of each variant following the final purification step. The anticipated molecular weight of each variant is ~38 kDa.

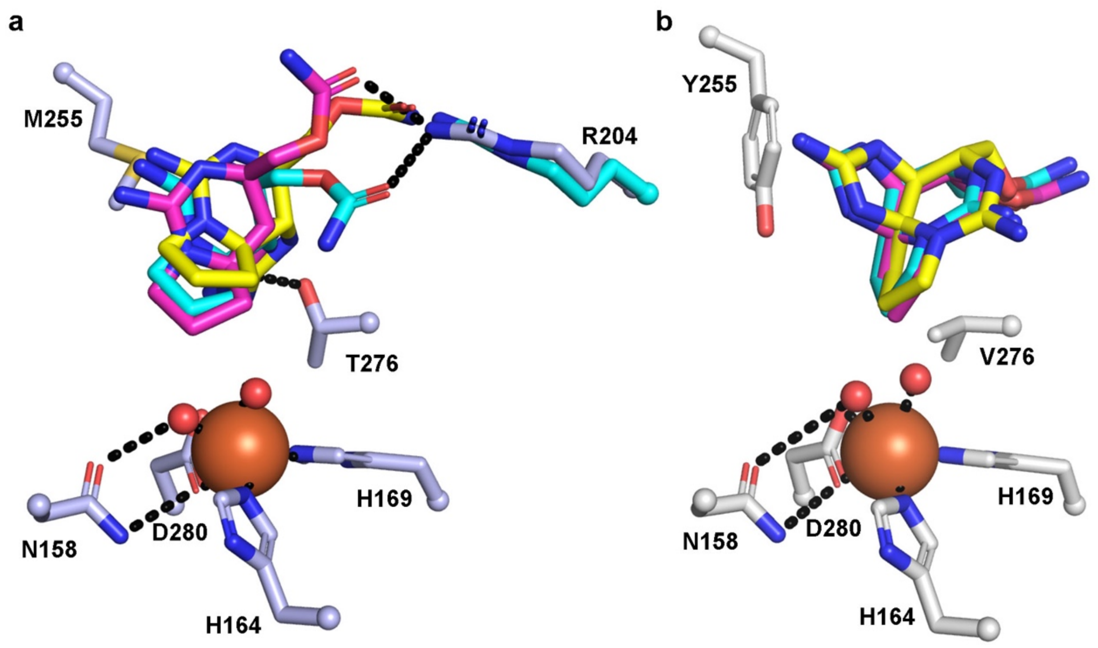

Supplementary Figure 3. ddSTX is bound to SxtT and GxtA in each monomeric unit of the trimer. (a) In SxtT, these orientations slightly differ in the position of the carbamate sidechains. The molecule of ddSTX found in chains A, B, and C are colored dark pink, cyan, and yellow, respectively. In chains A and B, the loop that spans the range 195-215 is ordered and Arg204 interacts with the substrate. In chain C, this loop is too disordered to be modeled. (b) In GxtA, the observed orientation of ddSTX is more consistent between chains. The molecule of ddSTX found in chains A, B, and C are colored dark pink, cyan, and yellow, respectively. The loop, despite being ordered in chain C, is found in the “open” conformation and does not interact with ddSTX.

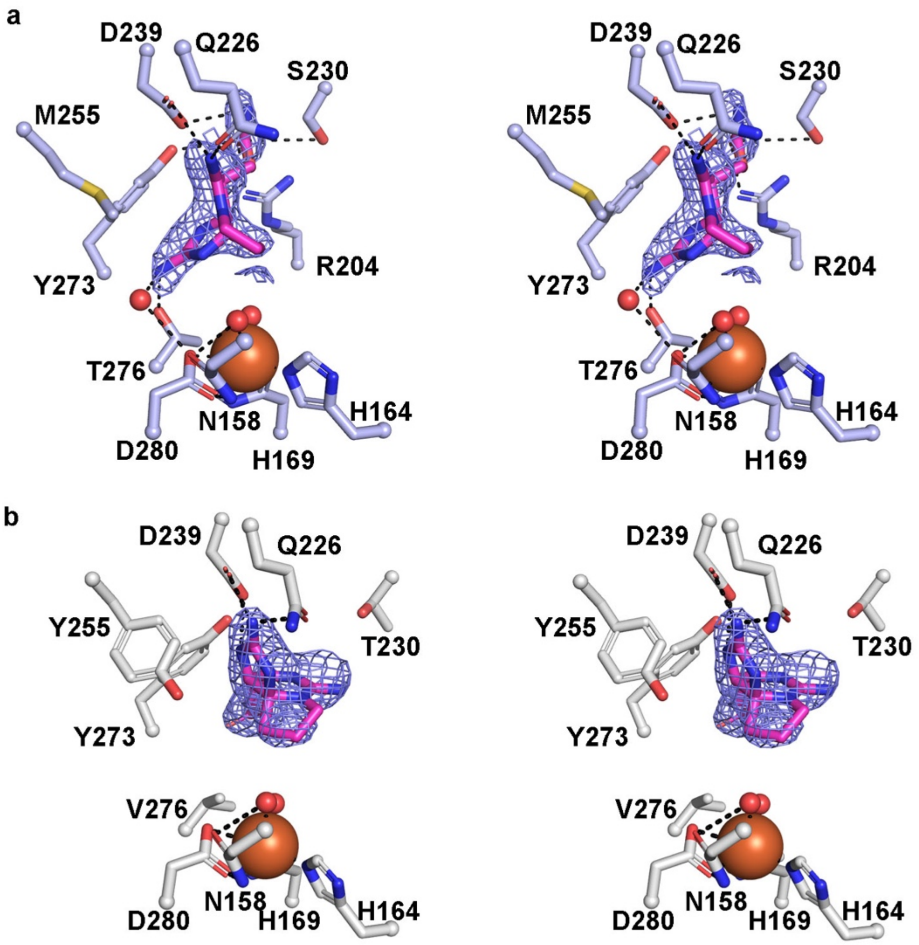

Supplementary Figure 4. ddSTX is bound in the active sites in SxtT and GxtA. (a) Stereo Figure of ddSTX bound to SxtT. Here, ddSTX interacts with Ser230, Tyr273, Asp239, Gln226, and Arg204. (b) Stereo figure of ddSTX bound to GxtA. In this structure, ddSTX forms interactions with Asp 239 and Gln226. Both panels are shown with 2Fo-Fc simulated annealing composite omit electron density maps contoured at 0.9σ.

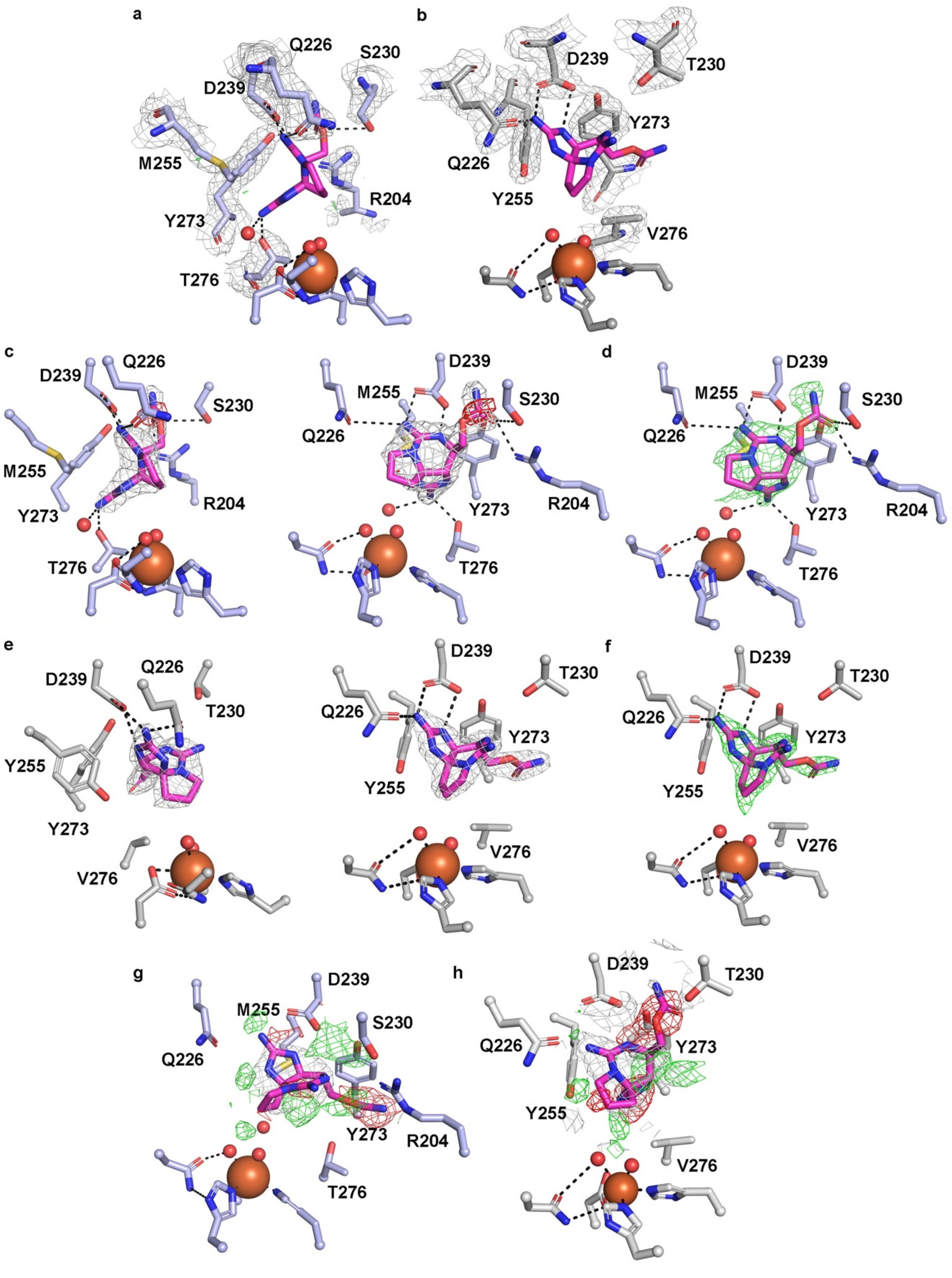

Supplementary Figure 5. ddSTX makes specific interactions in the active sites of SxtT and GxtA. (a) 2Fo-Fc and Fo-Fc electron density maps contoured at 1.0σ and ±3.0σ, respectively are shown for the sidechains of SxtT implicated in forming the ddSTX binding site. (b) Similar to panel a, this panel shows 2Fo-Fc and Fo-Fc electron density maps contoured at 1.0σ and ±3.0σ, respectively

around the sidechains of GxtA implicated in forming the ddSTX binding site. (c) 2Fo-Fc and Fo-Fc electron density maps contoured at 1.0σ and ±3.0σ, respectively around ddSTX in two orientations from chain A of SxtT. (d) An Fo-Fc omit electron density map calculated after ddSTX was omitted from the refined structure of SxtT. This map is contoured at 3.0σ around ddSTX. (e) 2Fo-Fc and Fo-Fc electron density maps contoured at 1.0σ and ±3.0σ, respectively around ddSTX in two orientations from chain A of GxtA. (f) An Fo-Fc omit electron density map calculated after ddSTX was omitted from the refined structure of GxtA. This map is contoured at 3.0 σ around ddSTX. (g) In this panel the orientation of ddSTX in GxtA has been refined in the active site of SxtT. This incorrect ddSTX orientation is shown with 2Fo-Fc and Fo-Fc electron density maps contoured at 1.0σ and ±3.0σ, respectively. (h) Similar to panel g, in this panel, the orientation of ddSTX from SxtT has been refined in the active site of GxtA. This incorrect orientation of ddSTX is shown with 2Fo-Fc and Fo-Fc electron density maps contoured at 1.0σ and ±3.0σ, respectively. In this figure, all 2Fo-Fc maps are shown in gray, whereas positive Fo-Fc difference density maps are shown in green and negative Fo-Fc difference density maps are shown in red.

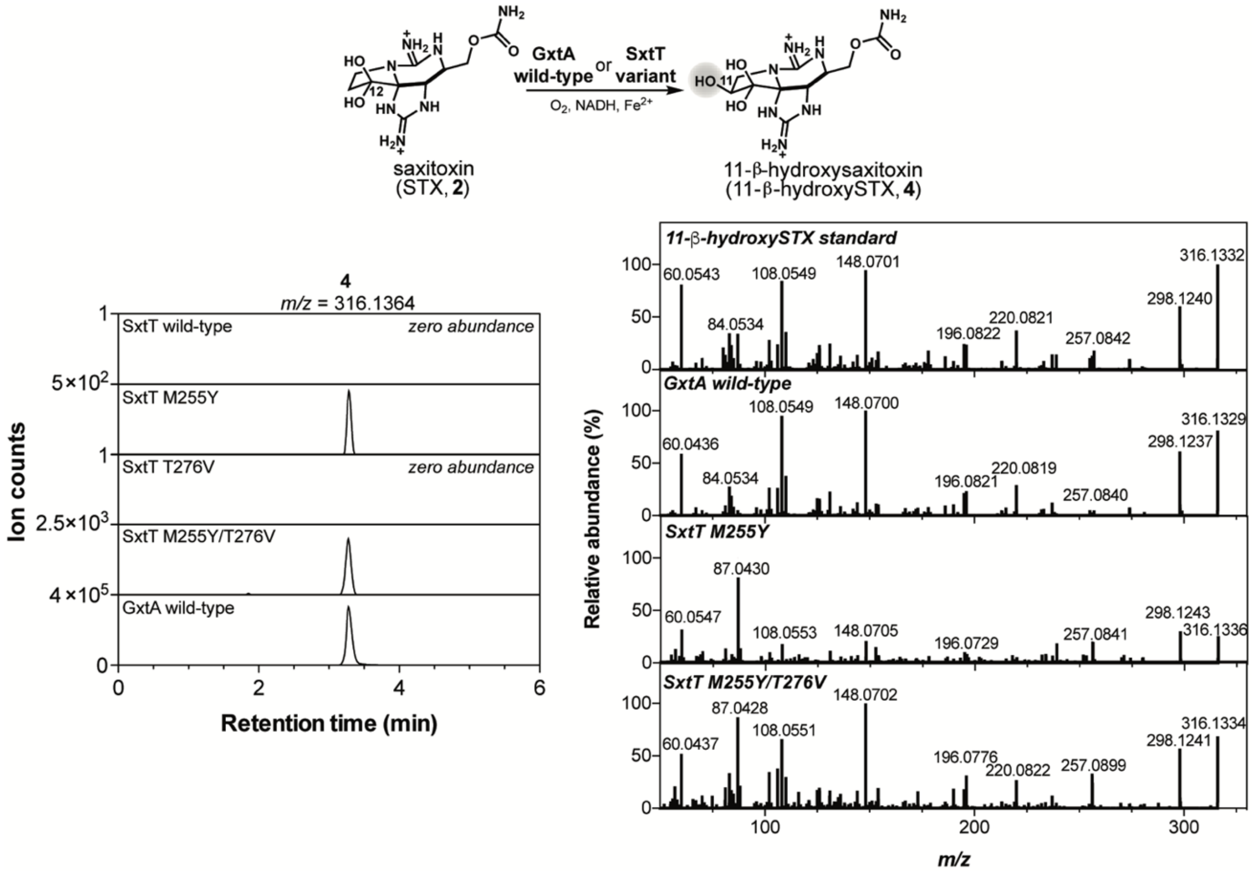

Supplementary Figure 6. As demonstrated using HILIC-MS and targeted MS/MS, similar to the native GxtA reaction, SxtT M255Y and SxtT double variant hydroxylate STX (**2**) at the C11 position to form 11- β-hydroxySTX (**4**).

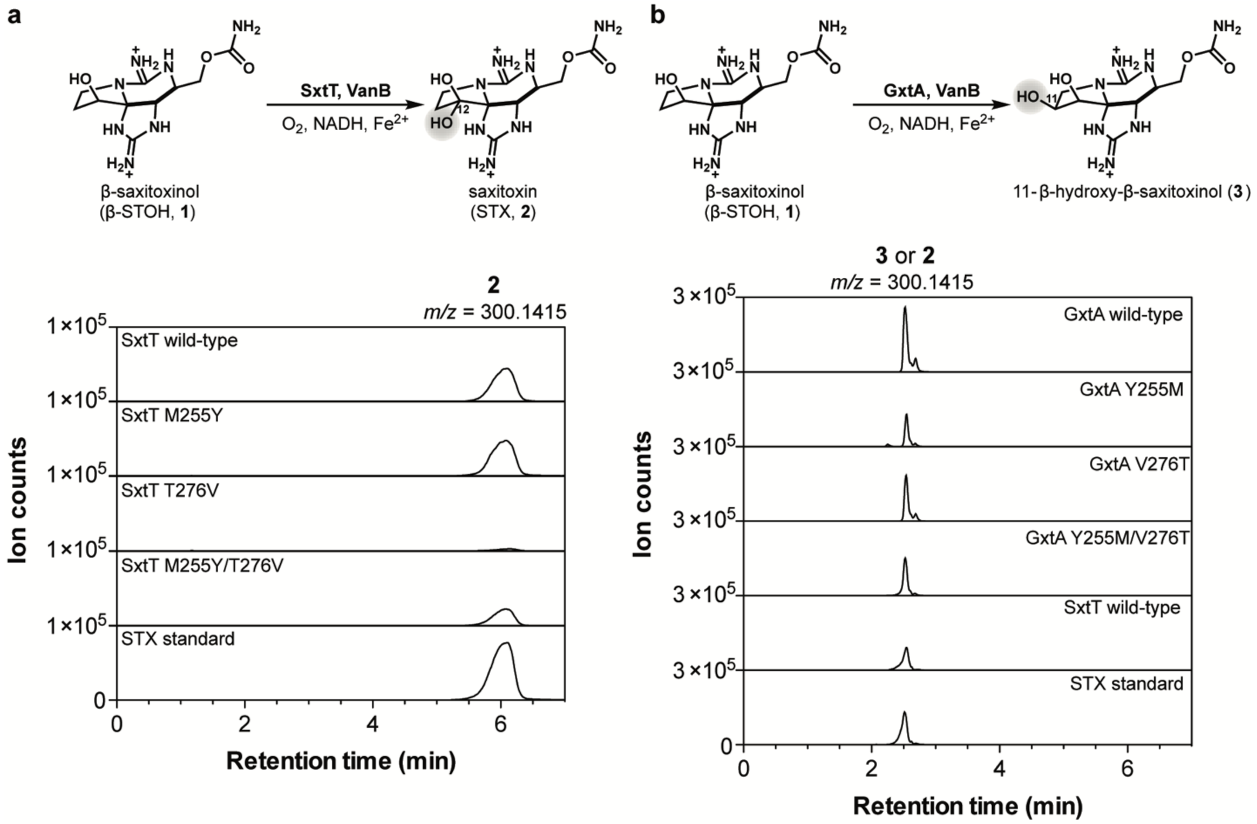

Supplementary Figure 7. Single active site variants of SxtT and GxtA maintain wild-type monohydroxlyation activity as demonstrated using HILIC-MS extracted ion chromatograms of the SxtT and GxtA reaction product(s) with β-STOH (**1**). *m/z* = 300.1415 represents the exact mass of both STX (**2**) and **3**. (a) SxtT wild-type and variants, analyzed using 15% A isocratic HILIC method. Scheme shown depicts wild-type reaction. (b) GxtA wild-type and variants, analyzed using 18% A isocratic HILIC method. Scheme shown depicts wild-type reaction.

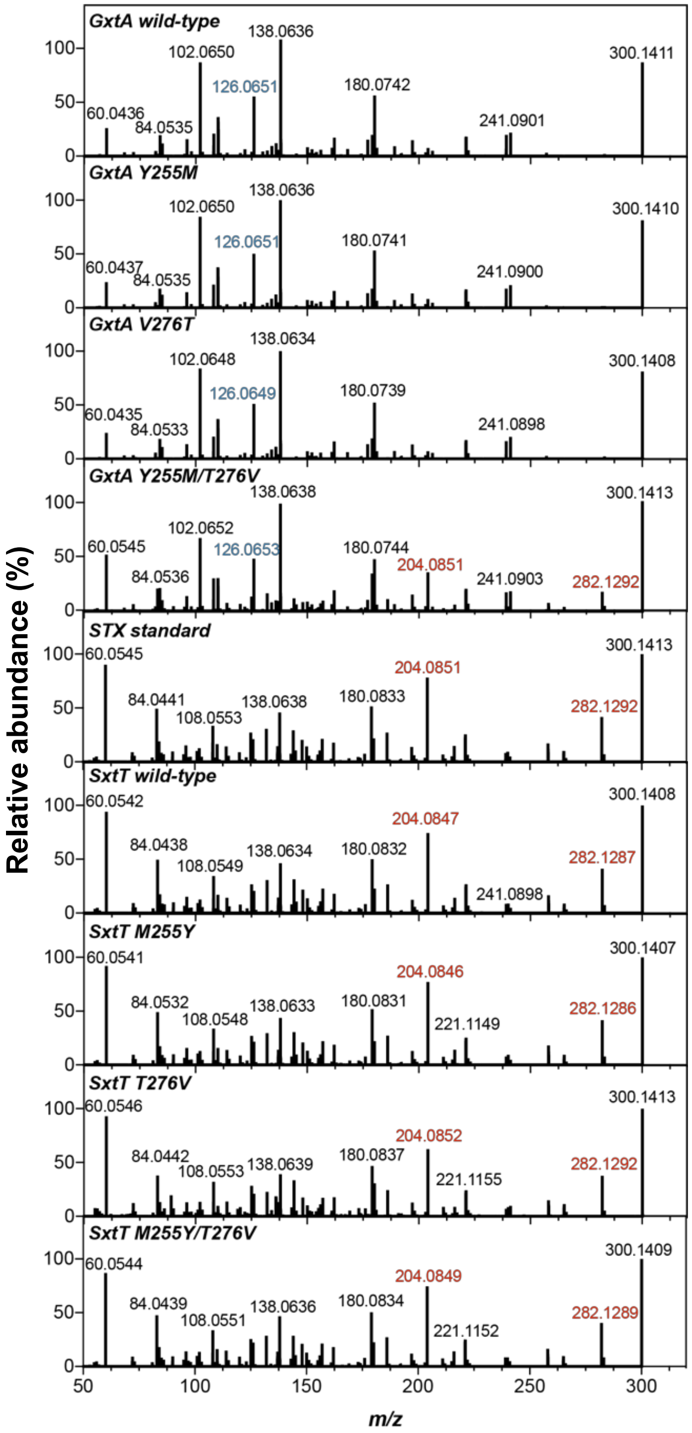

Supplementary Figure 8. Targeted MS/MS spectra of *m/z* = 300.1415 products from reactions with SxtT and GxtA variants with β-STOH (**1**). Numbers in red correspond to those that appear only when STX (**2**) is formed. Numbers in blue correspond to those that are prevalent only when **3** is formed. Reactions were performed as described in Methods Section IV and analyzed by HILIC-MS. The exact mass of 11-β-hydroxySTX (**4**) is [M+H] = 316.1336.

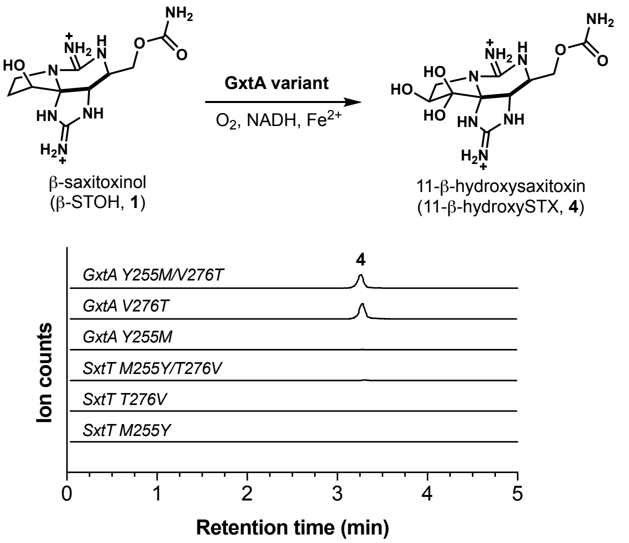

Supplementary Figure 9. The GxtA variant V276T is able to catalyze two hydroxylation reactions when provided β-STOH (**1**) as a substrate. Production of the dihydroxylated product, 11-β-hydroxySTX (**4**) is shown using HILIC-MS. None of the other protein variants tested in this work deviate from catalyzing a monohydroxylation reaction.

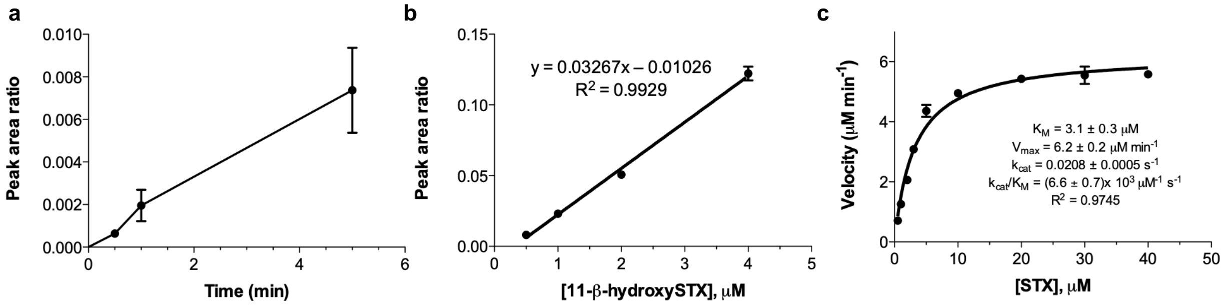

Supplementary Figure 10. GxtA steady-state kinetic analysis with STX (**2**). (a) Time course of 5 μM GxtA with 5 μM STX (**2**). (b) 11-β-hydroxySTX (**4**) standard curve used in the steady-state kinetic analysis. (c) Michaelis-Menten plot of GxtA reaction with STX. Data are presented as mean values +/- SD.

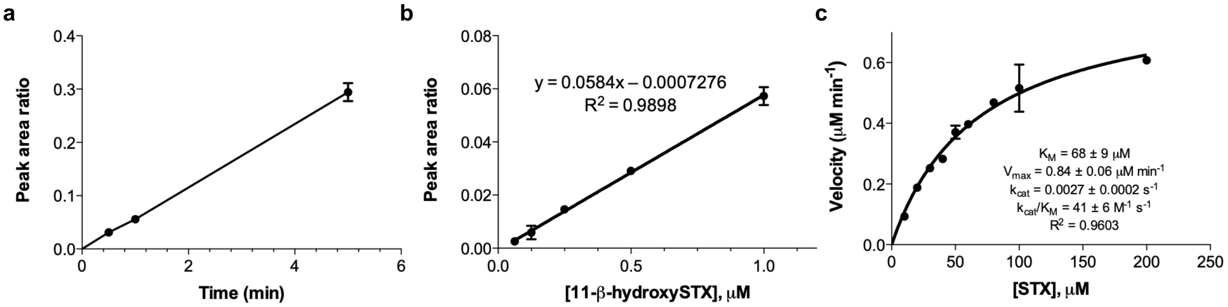

Supplementary Figure 11. SxtT M255Y/T276V steady-state kinetic analysis with STX (**2**). a) Time course of 5 μM SxtT M255Y/T276V with 50 μM STX (**2**). b) 11-β-hydroxySTX (**4**) standard curve used in the steady-state kinetic analysis. c) Michaelis-Menten plot of SxtT M255Y/T276V reaction with STX. Data are presented as mean values +/- SD.

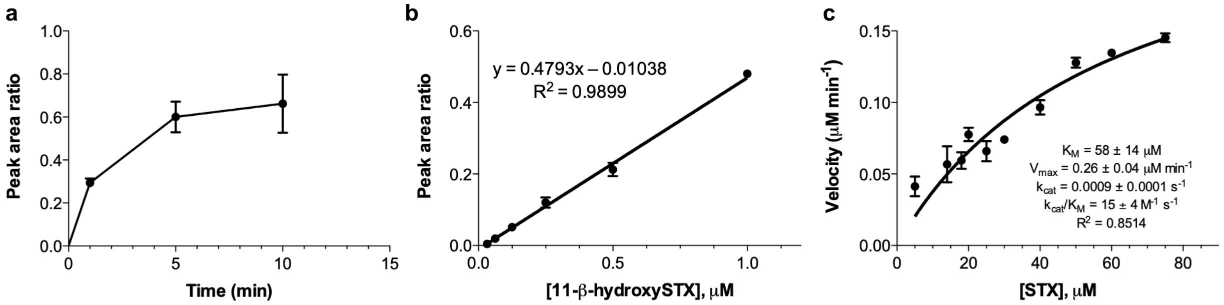

Supplementary Figure 12. SxtT M255Y steady-state kinetic analysis with STX (**2**). (a) Time course of 5 μM SxtT M255Y with 50 μM STX (**2**) in duplicate. (b) 11-β-hydroxySTX (**4**) standard curve used in the steady-state kinetic analysis. (c) Michaelis-Menten plot of SxtT M255Y reaction with STX. Data are presented as mean values +/- SD.

a

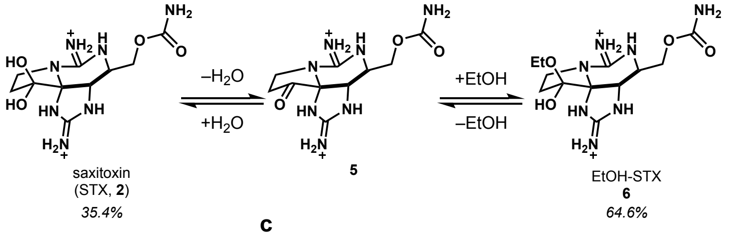

b

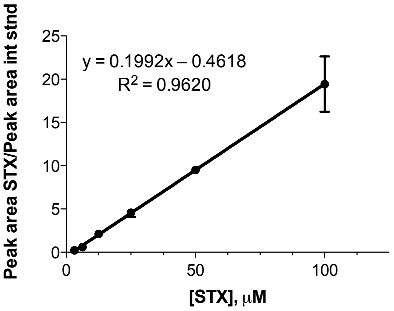

c

Trial	Peak area STX remaining	Peak area internal standard	Quantity STX remaining (μM)
**1**	2539449.3	176156.11	74.7
**2**	2704099.03	220412.48	63.9
**3**	2829411.92	198325.46	73.9
Average [STX] remaining			*70.8 ± 6.0 μM*
Average % STX in 200 μM			*35.4%*
Average % EtOH-STX in 200 μM			*64.6%*

Supplementary Figure 13. Extent of ethanol incorporation into STX (**2**) under our reaction conditions. (a) Scheme of STX (**2**) equilibrium in the presence of ethanol. (b) Standard curve of STX (**2**) used to quantify remaining STX (**2**) after incubation with ethanol. Data are presented as mean values +/- SD. (c) Raw data obtained from triplicate STX (**2**) incubations with ethanol and extrapolated percent EtOH-STX (**6**) present.

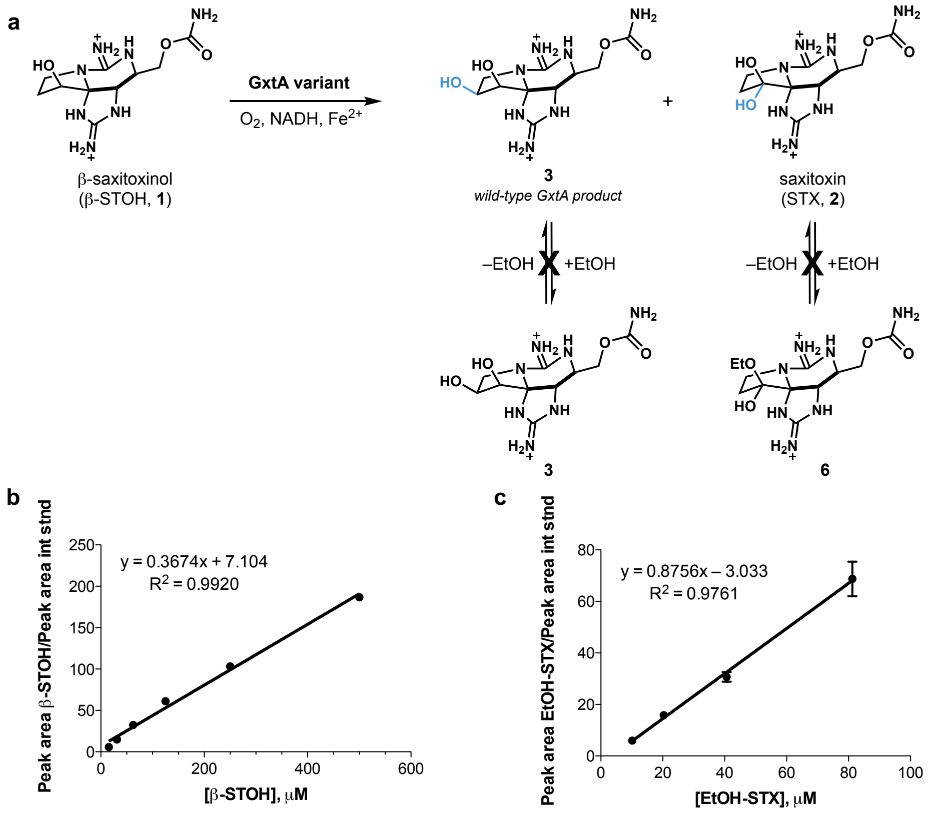

Supplementary Figure 14. GxtA Y255M/V276T reaction scheme and standard curves. (a) GxtA variant reaction scheme with β-STOH (**1**) and potential for incorporation of ethanol. (b) β-STOH (**1**) standard curve. (c) EtOH-STX (**6**) standard curve. Data are presented as mean values +/- SD.

Supplementary Table 1. Protein sequence accession numbers (GenBank), DNA sources, and vectors used in this study.

Protein	Organism of origin	Accession	DNA source	Vectors
SxtT	*Microseira wollei*	ACG63840.1	GeneArt, subcloned	pET151, pMCSG9
GxtA	*Microseira wollei*	ACG63835.1	IDT, fragment	pMCSG7, pMCSG9
VanB	*Pseudomonas aeruginosa*	NP_253592.1	IDT, fragment	pMCSG7

Supplementary Table 2. Site-directed mutagenesis primers used in this study.

Primer name	Primer DNA sequence
SxtT_M255Y	5' – GCGTTCTGCGTTATCTGATTATGTGG – 3'
SxtT_T276V	5' – CGAATATGATGAAGTGATCGAACAGGATATTC – 3'
GxtA_Y255M	5' – AGCGTTCTGCGTATGCTGATTATGTGGAATG – 3'
GxtA_V276T	5' – CAGATTATGATCAGACCATCGAAGAAGATATTC – 3'

Supplementary Table 3. Data collection and refinement statistics.

	SxtT	GxtA	SxtT + ddSTX	GxtA + ddSTX
**Data collection**				
Space group	*C*222	*P*12$_1$1	*C*222	*P*12$_1$1
Cell dimensions				
a, *b*, *c* (Å)	152.3, 159.6, 116.0	74.73, 96.93, 80.81	152.3, 159.6, 115.9	74.58, 96.98, 80.41
α, β, γ (°)	90, 90, 90	90, 107.0, 90	90, 90, 90	90, 107.2, 90
Resolution (Å)	30-1.86	40-2.20	30-2.10	40-2.18
R_{merge}	0.074 (0.907)	0.186 (0.477)	0.087 (0.436)	0.090 (0.755)
$I / \sigma I$	25.64 (2.36)	10.56 (1.56)	15.8 (2.07)	15.3 (1.84)
Completeness (%)	100 (100)	96.3 (90.9)	98.4 (89.0)	100 (100)
Redundancy	7.6 (7.5)	4.3 (2.2)	4.7 (3.4)	3.8 (3.8)
CC1/2	0996 (0.853)	0.981 (0.730)	0.984 (0.890)	0.995 (0.607)
**Refinement**				
Resolution (Å)	1.86	2.20	2.10	2.18
No. reflections	117619	54048	81773	56607
R_{work} / R_{free}	0.170, 0.198	0.192, 0.219	0.168, 0.207	0.171, 0.227
No. atoms				
Protein	8114	7759	8056	7721
[2Fe-2S]	12	12	12	12
Non-heme Fe	3	3	3	3
Glycerol	36	6	42	54
Sulfate	65	-	65	-
Water	885	359	443	298
ddSTX	-	-	57	57
B-factors				
Overall	40.65	43.81	45.23	39.16
Protein	39.55	43.77	44.72	38.87
ddSTX	-	-	65.34	50.75
[2Fe-2S]	28.54	30.80	32.46	28.13
Non-heme Fe	30.26	33.87	32.07	26.41
Glycerol	59.32	64.57	61.80	55.06
Sulfate	88.64	-	89.05	-
Water	46.54	49.31	46.28	40.56
R.m.s. deviations				
Bond lengths (Å)	0.008	0.012	0.008	0.008
Bond angles (°)	0.981	1.574	0.969	0.970

*Values in parentheses are for highest-resolution shell.

Supplementary Table 4. Summary of GxtA EtOH-incorporation results with β-STOH.

GxtA Variant	Trial	Quantity β-STOH remaining (μM)	Quantity EtOH-STX (μM)	Quantity 3 (μM)	Total % conversion	% conversion to 3	% EtOH-STX
Wild-type	1	430.0	0	70.0	14.0	14.0	0.0
Wild-type	2	327.1	0	172.9	34.6	34.6	0.0
Wild-type	3	357.6	0	142.4	28.5	28.5	0.0
Y255M	1	310.5	0.171	189.4	37.9	37.9	0.0
Y255M	2	324.2	0.171	175.6	35.2	35.1	0.1
Y255M	3	348.6	0.197	151.3	30.3	30.3	0.0
V276T	1	349.0	0.350	150.6	30.2	30.1	0.1
V276T	2	363.1	0.475	136.5	27.4	27.3	0.1
V276T	3	297.3	0.484	202.2	40.5	40.4	0.1
Y255M/V276T	1	385.5	9.110	105.3	22.9	21.1	1.8
Y255M/V276T	2	323.6	9.490	166.9	35.3	33.4	1.9
Y255M/V276T	3	359.5	9.933	130.6	28.1	26.1	2.0
